# Measuring inequality in quality of life: further evidence that the EQ-5D-5L may underestimate it

**DOI:** 10.1007/s11136-026-04294-w

**Published:** 2026-06-15

**Authors:** Admassu N. Lamu, Gang Chen, Ling Jie Cheng, Jan Abel Olsen

**Affiliations:** 1 A Lamu Consulting, Bergen, Norway; 2 Cancer Health Services Research Unit, University of Melbourne, Melbourne, Australia; 3 National Perinatal Epidemiology Unit, Nuffield Department of Women’s & Reproductive Health, University of Oxford, Oxford, United Kingdom; 4 Alice Lee Centre for Nursing Studies, Yong Loo Lin School of Medicine, National University of Singapore, Singapore, Singapore; 5 Department of Community Medicine, UiT The Arctic University of Norway, Tromsø, Norway

**Keywords:** EQ-5D-5L, EQ VAS, Health inequality, Socioeconomic gradient, EQ-DAPHNIE, Health-related quality of life

## Abstract

**Purpose:**

A previous study found that individuals with identical EQ-5D-5L profiles reported systematically higher EQ VAS scores with increasing educational attainment, which suggests a ‘hidden’ socioeconomic gradient not captured by the EQ-5D-5L. This study examines the robustness and generalisability of these findings using multi-country data.

**Methods:**

We analysed data from 32,327 respondents aged 25 to 79 years across eight high-income countries: Australia, Canada, France, Germany, the Netherlands, New Zealand, the UK, and the US. The data came from the EQ-DAPHNIE study. Within ten selected EQ-5D-5L health profiles, we used linear regression models to estimate the associations between EQ VAS scores and educational attainment or subjective income status, adjusting for age, sex, and country.

**Results:**

We observed a consistent educational gradient in EQ VAS scores across most EQ-5D-5L profiles. Tertiary education was associated with higher scores in all ten profiles, with effects statistically significant at *p* < 0.10 in seven, of which four at *p* < 0.01. Income status showed an even stronger gradient, with significant associations in nine of the ten profiles. These patterns were evident in all eight countries.

**Conclusion:**

These multi-country findings provide robust evidence of a socioeconomic gradient in EQ VAS scores among respondents who report identical EQ-5D-5L health profiles, over and above what is reflected in the five EQ-5D-5L dimensions. This pattern has implications for the use of EQ-5D-5L values in equity-informative health technology assessment and population health monitoring.

**Supplementary Information:**

The online version contains supplementary material available at https://doi.org/10.1007/s11136-026-04294-w.

## Introduction

Health-related quality of life instruments such as the EQ-5D-5L are widely used in health technology assessment, clinical trials, and population health monitoring [1]. They characterise health by asking respondents to report problems across a fixed set of dimensions: in the EQ-5D-5L, these are mobility, self-care, usual activities, pain or discomfort, and anxiety or depression. Respondents are also asked to rate their overall health on a visual analogue scale (EQ VAS). The five descriptive dimensions were selected to represent aspects of health that are common across populations, rather than to capture socioeconomic circumstances directly. Nevertheless, when respondents from different socioeconomic groups classify themselves in the same EQ-5D-5L health profile, any remaining systematic differences in their EQ VAS scores may provide insight into both the content coverage of the descriptive system and the way individuals use the response scales.

A longstanding literature in health economics and epidemiology has shown that self-reported health is susceptible to reporting heterogeneity: respondents with different characteristics may use the same response options in systematically different ways, even when their underlying health is similar [2, 3]. Analyses using anchoring vignettes, for example, show that correcting for reporting differences can materially change estimated health disparities by socioeconomic status [2, 3]. A complementary strand of work has examined whether the EQ-5D descriptive system omits dimensions relevant to Health-related quality of life, particularly psychosocial content, and has assessed the impact of candidate bolt-on items on content validity and discriminatory power [4–6]. Together, these strands of research raise the question of whether, within an identical reported EQ-5D-5L profile, EQ VAS scores may still vary systematically by socioeconomic position.

A recently published study showed that individuals with identical EQ-5D-5L profiles reported systematically higher EQ VAS scores with increasing educational attainment [7]. These findings suggest either that the use of EQ-5D-5L values may underestimate health inequalities or that a hidden socioeconomic gradient exists that the EQ-5D-5L descriptive system does not capture. However, that study relied on Norwegian data from the Tromsø Study, conducted in a relatively small city [8], and on a multi-country survey, the MIC study, in which approximately 80% of participants had chronic conditions [9]. These limitations highlight the need for evidence from larger and more diverse populations to assess the robustness and generalisability of the observed pattern.

Against this background, we replicate and extend the analytic approach of Olsen et al. [7] using a much larger multi-country dataset, the EQ-DAPHNIE survey [1]. In addition to educational attainment, we use *subjective income status* as an alternative indicator of socioeconomic position, motivated by growing evidence that perceived income adequacy captures both economic circumstances and perceived social standing in ways not fully reflected by objective income [10]. We assess robustness in two further ways that were not feasible in the earlier study: by examining the distribution of EQ VAS scores within each profile and by conducting country-stratified analyses for the two profiles with sufficient sample sizes across all eight countries.

Therefore, this paper addresses two research questions:

I. Is there evidence of a ‘hidden’ education–health gradient among respondents who report identical EQ-5D-5L health profiles?
II. Does a similar pattern emerge when an alternative socioeconomic indicator, such as income status, is used?

## Methods

### Data and study design

We used data from the EuroQol Data for Assessment of Population Health Needs and Instrument Evaluation (EQ-DAPHNIE) study, a cross-sectional online survey of the general adult population aged 18 years and older. We included eight high-income countries: Australia, Canada, France, Germany, the Netherlands, New Zealand, the UK, and the US. Participants were recruited through the online panel provider Dynata (http://www.dynata.com/). In each country, the target sample was approximately 4500 respondents. Quota sampling was used to improve representativeness by age, sex, income, region, and language [1].

We applied several procedures to ensure data quality and validity across countries, including confirmation of informed consent, consistency checks using duplicated or similar questions, and minimum completion time thresholds [1, 11]. In line with previous work [7], we excluded respondents younger than 25 years, the expected age of education completion, and those aged over 80 years. We further excluded respondents with implausibly low body mass index (BMI) values as an additional data-quality check; only observations with BMI > 15 kg/m^2^ were retained. After applying all of these data editing procedures, 32,327 observations remained for the final analyses.

### Variables

Educational attainment was reported using country-specific categories and harmonised into three levels to enable cross-country comparability: low (primary or secondary), medium (post-secondary non-tertiary or equivalent), and high (tertiary education, including bachelor’s degree or higher). This classification is consistent with the harmonisation used by Olsen et al. [7] and facilitates comparability across the eight countries included in this analysis.

As an alternative indicator of socioeconomic position, we used subjective income status to capture perceived financial adequacy. This measure is particularly relevant in cross-country analyses, in which absolute income levels and purchasing power differ substantially [12–14]. Income status was assessed by asking respondents how they felt about their household’s income nowadays, with four response options: comfortable, coping, difficult, and very difficult. Because relatively few respondents selected the lowest category, we combined the last two categories.

For comparability with our previous study [7], we examined variation in EQ VAS scores within the same 10 EQ-5D-5L profiles: 11111, 11121, 11122, 21121, 11112, 11221, 11131, 11132, 21231, and 11123. Two of these profiles, 21121 and 11221, were less prevalent in the current dataset.

Health-related quality of life was measured using the EQ VAS, which ranges from 0 (worst imaginable health) to 100 (best imaginable health). To address potential inconsistencies between EQ VAS and EQ-5D-5L responses, we excluded EQ VAS scores below 50 for the full-health profile (11111) and below 30 for all other profiles (Supplementary Table S1). These thresholds follow Olsen et al. [7] and were intended to remove a small number of responses that were internally inconsistent with the reported EQ-5D-5L profile. Specifically, an EQ VAS score below 50 was considered implausible for respondents reporting full health (11111), and a score below 30 was considered implausible for any of the very mild profiles examined here. The proportion of excluded cases was small, ranging from 0.6 to 3.1% across profiles (Supplementary Table S1). Further details of the selected health profiles and the exclusion procedure have been reported elsewhere [7].

### Analyses

Before fitting the regression models, we examined the distribution of EQ VAS scores within each of the 10 selected EQ-5D-5L profiles. We computed descriptive statistics, including the mean, standard deviation, median, first and third quartiles, and interquartile range, and inspected combined violin and box plots to assess within-profile skewness and the bounded nature of the EQ VAS scale.

After exploring both linear and alternative semiparametric regression approaches, we found that linear regression models produced results similar to those from more complex specifications, as reported elsewhere [7]. Given their simplicity and ease of interpretation, we used linear regression models with survey weights to account for the complex sampling design.

For each EQ-5D-5L profile, we estimated separate regression models with EQ VAS as the dependent variable. Educational attainment was the primary explanatory variable, and models were adjusted for age, sex, and country fixed effects. We conducted corresponding analyses using subjective income status as an alternative indicator of socioeconomic position.

To complement the pooled analyses and assess whether the socioeconomic gradient was also present within countries, we conducted country-stratified regressions for the two profiles with sufficient sample sizes across all eight countries: the full-health profile (11111) and the mildly affected profile (11121). For each country, we fitted separate linear regressions of EQ VAS on educational attainment and, in a parallel specification, on subjective income status, adjusting for age and sex.

Missing data were limited, with the highest proportion observed for EQ VAS (5.8%), followed by educational attainment (2.0%). Assuming that data were missing at random, we used Full Information Maximum Likelihood estimation in the regression analyses. Under this assumption, this approach yields unbiased parameter estimates [15]. Following the convention adopted in the parent study [7], we report statistical significance at three levels: *p* < 0.10, *p* < 0.05, and *p* < 0.01.

## Results

Table 1and Supplementary Table S1 present respondent characteristics and the distribution of the most prevalent EQ-5D-5L health profiles. A substantial proportion of respondents had attained tertiary education (44.0%) and reported coping with their current income (40.7%). The 10 selected EQ-5D-5L profiles covered 61% of respondents.

**Table 1:** Sample characteristics: EQ-DAPHNIE in eight countries, aged 25–79 years

Variable	*n*	Percent	Mean EQ VAS	SD
Total sample	32,327	100.0	72.9	19.6
*Sex*				
Female	17,483	55.0	71.8	19.9
Male	14,490	45.0	74.2	19.0
*Age category*				
25–40 years	10,405	32.2	74.3	19.0
41–60 years	11,714	36.2	70.9	20.4
61–79 years	10,189	31.5	73.7	18.9
*Educational attainment*				
Low	10,107	32.0	69.0	21.3
Medium	7573	24.0	71.9	19.8
High	14,006	44.0	76.1	17.4
*Income status*,* n (%)*				
Difficult	9559	30.0	64.4	21.9
Coping	13,167	41.0	73.8	17.7
Comfortable	9467	29.0	80.1	15.9
*Country*				
Australia	4647	14.0	72.7	19.5
Canada	3952	12.0	71.0	19.4
France	3953	12.0	72.3	19.6
Germany	3940	12.0	73.4	20.3
Netherlands	3927	12.0	74.4	18.5
New Zealand	4013	12.0	75.5	18.2
United Kingdom	4010	12.0	70.6	21.2
United States	3885	12.0	73.2	19.1

Consistent with the previous study, a minimum age of 25 years was chosen to account for the completion of university-level education. Because of variation in the coding across countries, educational attainment was harmonised into three groups: *Low* (primary or secondary school), *Medium* (post-secondary non-tertiary or equivalent), and *High* (all forms of tertiary education from bachelor’s degree and above). *SD*: standard deviation. Row totals may not sum exactly to the total sample size because of missing values on individual variables; percentages are calculated on non-missing cases

**Fig. 1:**
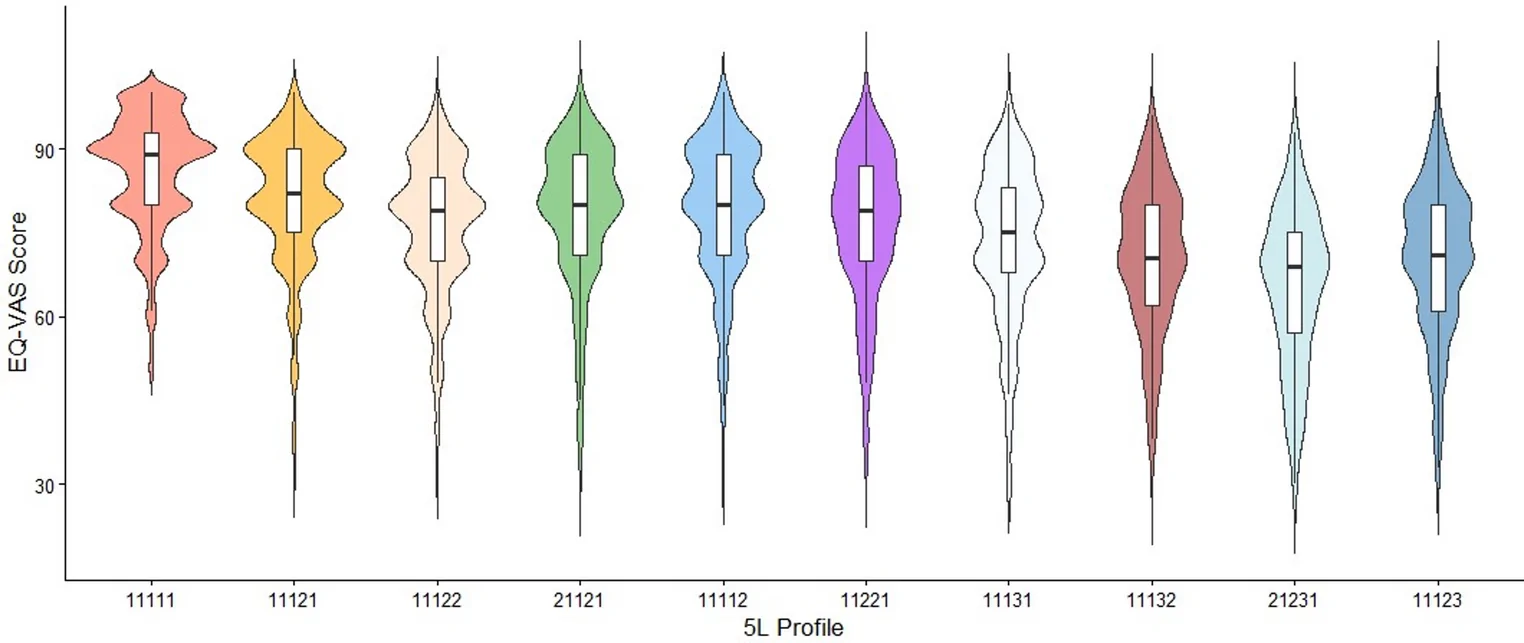
Distribution of EQ VAS scores by EQ-5D-5L profile: combined violin and box plot. *Note*: Violin plots show the kernel density estimate of the EQ VAS score distribution within each EQ-5D-5L profile; the width at each EQ VAS value is proportional to the density. Embedded box elements show the median (thick black line), interquartile range (white box), and the remainder of the distribution (thin grey line). The median EQ VAS score for the full-health profile (11111) is higher than for all other profiles, and within-profile distributions are concentrated around the median with mild left-skew consistent with the upper-bounded nature of the EQ VAS. Alternative plots (box plot only; violin and box plot with outliers highlighted) are presented in Supplementary Figs. S1 and S2

Table 2 shows the distribution of EQ VAS scores within each of the 10 EQ-5D-5L profiles. Median EQ VAS scores declined in the expected order across profiles of increasing severity, from 89 (IQR 80–93) in full health (11111) to 69 (IQR 57–75) in the most affected profile (21231). Within-profile distributions were concentrated around the median, with mild left skew, consistent with the upper-bounded nature of the EQ VAS (Fig. 1).

**Table 2:** Distribution of EQ VAS scores within each selected EQ-5D-5L profile

EQ-5D-5L profile	*n*	Mean	SD	Median	Q1	Q3	IQR
11111	8569	86.1	10.3	89.0	80.0	93.0	13.0
11121	3323	81.1	11.6	82.0	75.0	90.0	15.0
11122	2435	76.3	12.2	79.0	70.0	85.0	15.0
21121	689	78.5	12.8	80.0	71.0	89.0	18.0
11112	2289	79.1	12.6	80.0	71.0	89.0	18.0
11221	307	76.5	13.1	79.0	70.0	87.0	17.0
11131	444	73.2	13.8	75.0	68.0	83.0	15.0
11132	355	70.6	12.9	70.5	62.0	80.0	18.0
21231	218	65.1	13.9	69.0	57.0	75.0	18.0
11123	869	70.3	13.2	71.0	61.0	80.0	19.0

Sample restricted to respondents aged 25–79 years, with body mass index (BMI) > 15 kg/m^2^ and after applying the EQ VAS exclusions described in the Methods (EQ VAS < 50 for profile 11111 and < 30 for all other profiles). *SD*: standard deviation; *Q1*: first quartile; *Q3*: third quartile; *IQR*: interquartile range. Within-profile distributions are concentrated around the median, with left-skew consistent with the upper-bounded nature of the EQ VAS

Regression results (Table 3) showed clear socioeconomic gradients in EQ VAS scores across most profiles. Within profile 11121, EQ VAS scores were 0.863 points higher among respondents with medium education and 2.135 points higher among those with high education than among those with low education (Panel A). In the same profile, EQ VAS scores were 2.451 points higher among respondents coping with their income and 4.969 points higher among those living comfortably than among those reporting financial difficulties (Panel B). Overall, higher education was associated with higher EQ VAS scores in all 10 profiles, with seven associations statistically significant at *p* < 0.10, including four at *p* < 0.01, while significant income gradients were observed in nine of the 10 profiles.

**Table 3:** Linear regressions of EQ VAS scores on educational attainment and income status within selected EQ-5D-5L profiles, aged 25–79 years

Variables	Most prevalent profiles						Moderate-level profiles				
	11111	11121	11122	21121	11112	11221	11131		11132	21231	11123
***Panel A: Education gradient in EQ VAS***											
*Education (ref = Low)*											
Medium	1.045***	0.863	0.467	0.381	1.507*	3.419*	2.443		− 0.107	1.452	1.684
(SE)	(0.352)	(0.558)	(0.746)	(1.246)	(0.812)	(1.893)	(1.678)		(1.972)	(2.190)	(1.284)
High	1.554***	2.135***	1.183*	0.175	2.125***	6.489***	3.028*		0.815	4.753*	0.900
(SE)	(0.295)	(0.514)	(0.628)	(1.213)	(0.674)	(2.019)	(1.719)		(1.806)	(2.508)	(1.141)
Male (ref = Female)	− 0.659***	− 1.238***	0.084	0.080	− 0.470	− 0.802	0.466		− 0.039	0.378	− 1.204
(SE)	(0.234)	(0.414)	(0.528)	(1.007)	(0.564)	(1.515)	(1.342)		(1.519)	(1.872)	(0.968)
Constant	75.180***	73.913***	83.687***	47.336***	69.145***	73.799***	67.387***		74.819***	72.616***	70.195***
(SE)	(1.060)	(1.197)	(0.560)	(6.239)	(4.132)	(3.049)	(3.380)		(1.253)	(2.072)	(3.478)
R-squared	0.014	0.025	0.023	0.027	0.029	0.013	0.118		0.050	0.035	0.075
***Panel B: Income status gradient in EQ VAS***											
*Income (ref = Difficult)*											
Coping	1.840***	2.451***	2.125***	3.959***	1.632**	0.764	3.780**		2.917*	5.562**	0.355
(SE)	(0.352)	(0.566)	(0.586)	(1.368)	(0.636)	(1.975)	(1.654)		(1.558)	(2.199)	(0.994)
Comfortable	4.618***	4.969***	4.866***	4.284***	3.149***	2.320	5.283***		1.610	8.485***	2.928**
(SE)	(0.345)	(0.592)	(0.721)	(1.450)	(0.749)	(2.264)	(1.869)		(2.144)	(2.471)	(1.403)
Male (ref = Female)	− 0.925***	− 1.353***	− 0.218	0.020	− 0.612	− 0.770	0.116		− 0.083	0.299	− 1.259
(SE)	(0.231)	(0.411)	(0.526)	(0.998)	(0.564)	(1.548)	(1.337)		(1.516)	(1.839)	(0.967)
Constant	75.207***	73.545***	82.212***	46.390***	73.812***	71.554***	66.740***		75.384***	73.051***	71.193***
(SE)	(1.029)	(1.114)	(0.548)	(5.930)	(4.087)	(3.038)	(3.216)		(1.185)	(1.965)	(3.317)
Observations	8,569	3,323	2,435	689	2,289	307	444		355	218	869
R-squared	0.031	0.040	0.039	0.031	0.040	0.017	0.151		0.020	0.071	0.084

Entries are linear regression coefficients with robust standard errors in parentheses. Each column is a separate regression for the indicated EQ-5D-5L profile. Dependent variable: EQ VAS score. Panel A reports the model with educational attainment as the primary explanatory variable; Panel B reports the corresponding model with subjective income status. Both models control for age and country dummies (Australia as reference; Canada, France, Germany, the Netherlands, New Zealand, the UK, and the US). Sample sizes and R-squared values apply to both panels within a column and are reported once in Panel B. Educational attainment was harmonised into three levels: *Low* (primary or secondary; reference), *Medium* (post-secondary non-tertiary or equivalent), and *High* (tertiary, bachelor’s degree or higher). Income status: *Difficult* (reference; combined the two lowest categories), *Coping*, *Comfortable*. Significance: * *p* < 0.10; ** *p* < 0.05; *** *p* < 0.01

**Fig. 2:**
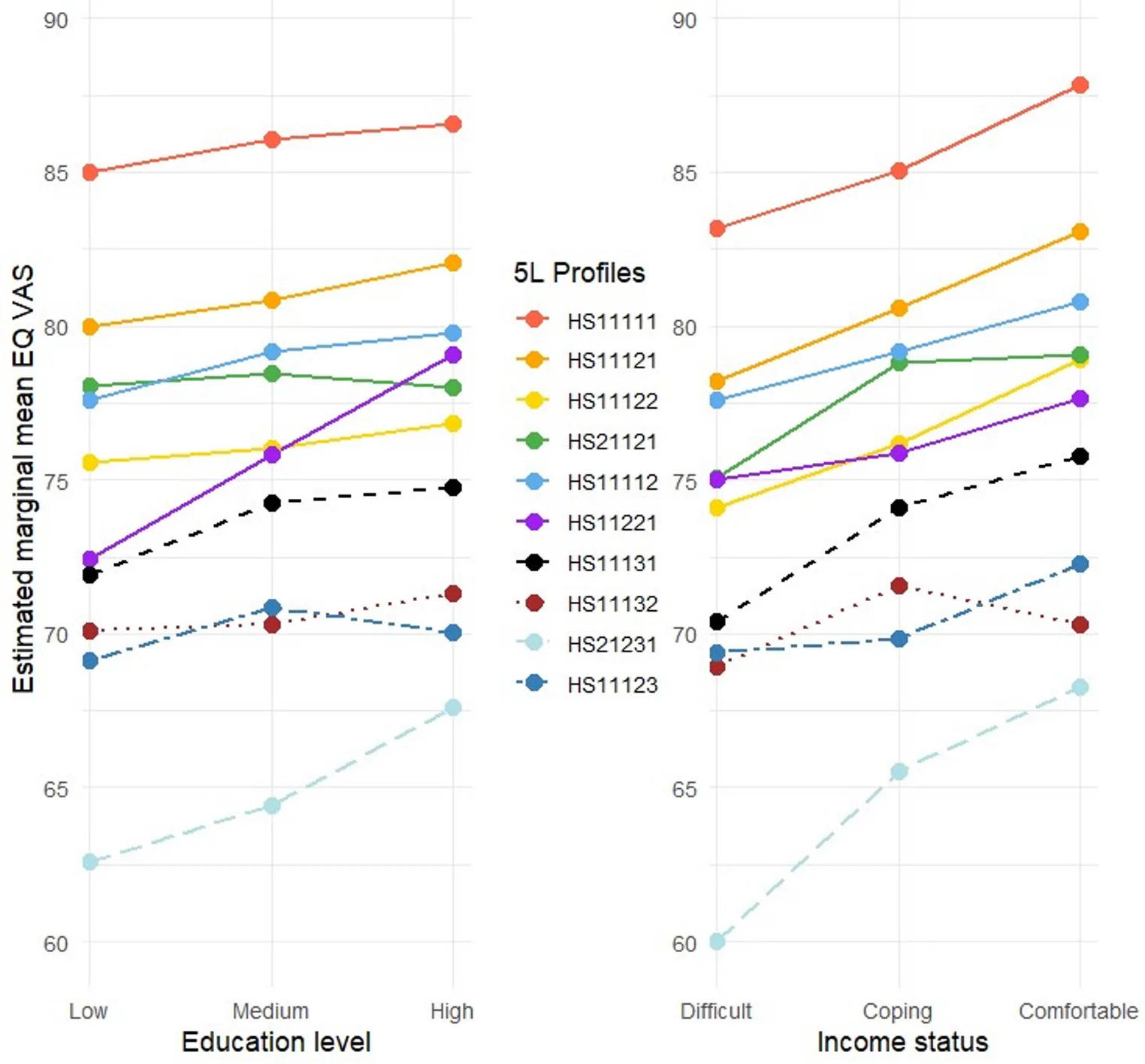
Mean EQ VAS by educational attainment and income status within each EQ-5D-5L profile, adjusted for age, sex and country. *Note*: Points are predictive margins from the linear regression models reported in Table 3, showing the mean EQ VAS score within each EQ-5D-5L profile by level of educational attainment (left panel) or income status (right panel), adjusted for age, sex and country dummies. Educational attainment was harmonised into three levels: *Low* (primary or secondary), *Medium* (post-secondary non-tertiary or equivalent), and *High* (tertiary education, bachelor’s degree or higher)

**Fig. 3:**
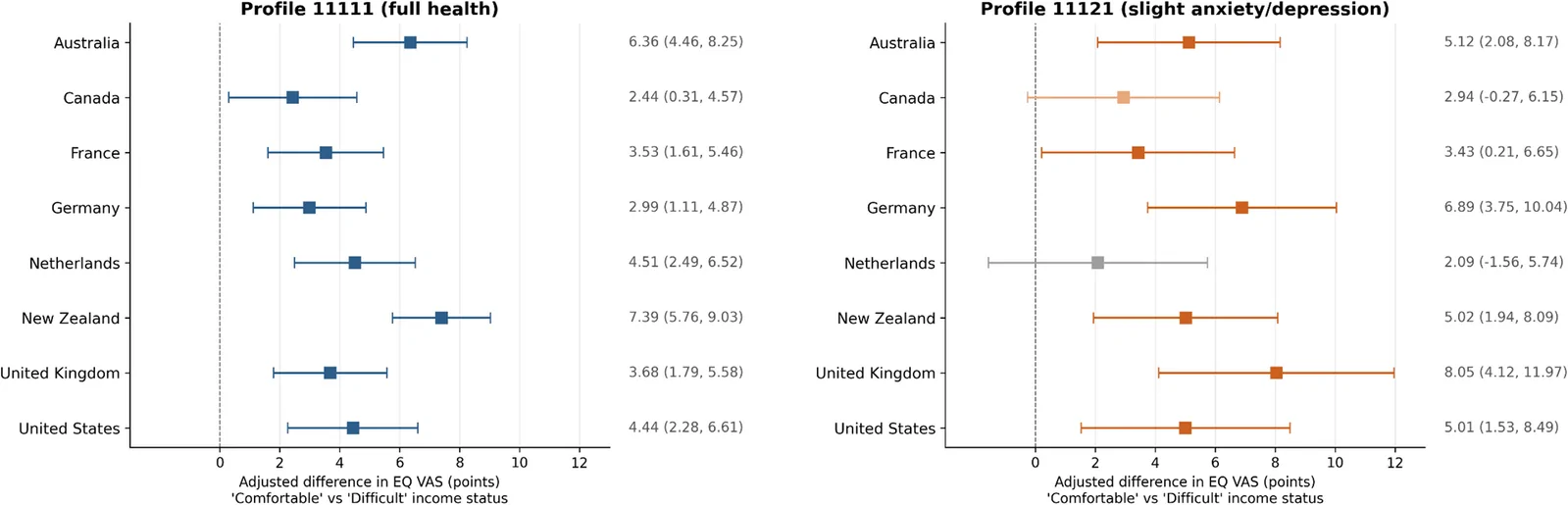
Country-stratified income gradient in mean EQ VAS within EQ-5D-5L profiles 11111 and 11121, adjusted for age and sex. *Note*: For each country, the plotted effect is the linear-regression coefficient on *Comfortable* income status (reference: *Difficult*), with 95% CIs (coefficient ± 1.96 × robust SE). Full-colour markers indicate *p* < 0.05; lighter orange (Canada, profile 11121) indicates *p* < 0.10 only; grey (Netherlands, profile 11121) indicates non-significant. Positive coefficients in all eight countries indicate a consistent within-profile income gradient. Full results in Supplementary Tables S2 and S3

Figure 2 presents predictive margins based on Table 3 and shows that these gradients were evident across nearly all profiles for both socioeconomic indicators. The only exception was a small non-significant decline for education in profile 11132. The gradients were steeper for subjective income status than for educational attainment. Country-stratified analyses were restricted to the two profiles (11111 and 11121) that had sufficient sample sizes in all eight countries; results are reported in Supplementary Tables S2 (profile 11111) and S3 (profile 11121), and the adjusted income gradient is summarised in Fig. 3. The income gradient was consistent across countries: for profile 11111, the coefficient for comfortable versus difficult income status was positive and statistically significant at *p* < 0.05 in all eight countries; for profile 11121, the corresponding coefficient was positive in all eight countries and statistically significant in seven at *p* < 0.10 (six at *p* < 0.05). By contrast, the education gradient was more heterogeneous in direction and magnitude across countries.

## Discussion

This multi-country analysis replicates and extends earlier evidence [7] that EQ VAS scores are systematically associated with socioeconomic position among respondents who report identical EQ-5D-5L health profiles. In a sample of more than 32,000 adults from eight high-income countries, both educational attainment and subjective income status showed clear positive gradients in EQ VAS scores across most of the 10 profiles examined. The gradient for subjective income status was stronger and more consistent than that for education. This pattern was also robust in country-stratified analyses of the two profiles for which such analyses were feasible. Together, these findings support earlier results from a single Norwegian city [7, 8] and from a multi-country survey with a high prevalence of chronic conditions [9].

Two broad, non-mutually exclusive explanations are consistent with these findings. First, the five EQ-5D-5L descriptive dimensions may not capture all aspects of health that respondents consider when rating their overall health on the EQ VAS. Dimensions such as vitality, sleep, social relationships, and community connectedness, which have been proposed as psychosocial bolt-ons to the EQ-5D, may be unequally distributed by socioeconomic position [4–6]. Under this interpretation, respondents with lower socioeconomic status who classify themselves in the same EQ-5D-5L state as their higher-SES peers may nevertheless experience more problems in domains not captured by the descriptive system, which are then reflected in lower EQ VAS scores. Second, the findings may reflect reporting heterogeneity: individuals from different socioeconomic groups may use the EQ VAS response scale in systematically different ways, as has been shown for several self-rated health measures using anchoring vignettes [2, 3]. Although our data cannot distinguish between these mechanisms, both point to the same practical conclusion: EQ-5D-5L descriptive responses alone do not fully summarise socioeconomic differences in overall self-rated health.

The stronger gradient for subjective income status than for education may reflect the fact that perceived income adequacy captures not only material circumstances but also perceived social standing, both of which are independently associated with health outcomes [10]. It may also be more directly comparable across countries than either absolute income or nationally coded education categories [12–14]. This interpretation is consistent with the greater cross-country stability of the income gradient observed in our analyses.

These findings have two main implications for the use of EQ-5D-5L values. First, in population health monitoring and equity analysis, EQ-5D-5L values are likely to understate socioeconomic disparities in overall self-rated health relative to analyses based on the EQ VAS, and both measures may therefore be worth reporting. Second, in equity-informative health technology assessment, including distributional cost-effectiveness analysis [16], the finding that respondents within the same EQ-5D-5L profile still differ systematically in EQ VAS score by socioeconomic position suggests that equity-weighted QALY calculations based on EQ-5D-5L values may miss part of the distributional signal. This does not undermine the use of the EQ-5D-5L in HTA. The descriptive system was not designed to measure socioeconomic circumstances, and its role within a deliberative HTA framework remains well established [16]. Rather, our findings strengthen the case for combining EQ-5D-5L values with complementary measures, such as the EQ VAS, selected bolt-on dimensions, or direct indicators of socioeconomic position, when equity is central to the decision problem.

## Strengths and limitations

Our study has several strengths. It uses a large multi-country sample recruited with quota sampling and subject to data-quality controls [1, 11]. The analytic design closely mirrors the earlier study, which facilitates comparison [7]. It also adds distributional and country-stratified analyses, which strengthen the robustness and generalisability of the conclusions.

Several limitations should be considered. First, the cross-sectional design precludes causal inference. We cannot determine whether lower socioeconomic status leads to poorer self-assessed health or whether unmeasured confounders influence both. Second, recruitment through an online panel may have introduced selection bias, because internet users may differ systematically from the general population in ways related to socioeconomic status and health reporting patterns. Third, perceived income adequacy is a subjective measure and may share psychological determinants with EQ VAS responses, such as optimism, which could inflate the observed associations. The stronger gradient for income status than for education may therefore partly reflect shared measurement properties. Fourth, EQ VAS may be more susceptible to response-style biases associated with socioeconomic position, and our study design cannot distinguish reporting differences from genuine health differences. Fifth, restricting the analysis to eight high-income countries limits generalisability to low- and middle-income settings. Sixth, the country-stratified analyses had limited precision for some estimates. Although the income gradient was directionally consistent across all eight countries for both profiles examined, statistical significance for the moderate profile (11121) was sensitive to the threshold used: the comfortable-versus-difficult income coefficient reached *p* < 0.05 in six countries, was marginally significant at *p* < 0.10 in Canada, and was not statistically significant in the Netherlands. Larger country-specific samples would help to clarify the precision of these estimates. Finally, we adopted the EQ VAS exclusion thresholds from Olsen et al. [7] to remove internally inconsistent responses. These thresholds should be interpreted as a logical consistency filter rather than a robustness choice: an EQ VAS score below 50 for respondents reporting full health (11111), or below 30 for any of the very mild profiles, is incompatible with the self-reported EQ-5D-5L profile and most plausibly reflects a response error. Retaining such responses would allow a small tail of internally inconsistent values to distort the within-profile mean and attenuate the estimated gradient. The number of affected cases was small (0.6 to 3.1% per profile; Supplementary Table S1), and applying the same rule preserves direct comparability with the published benchmark study.

## Conclusion

This study provides robust cross-national evidence of a socioeconomic gradient in overall self-rated health, measured by EQ VAS, among respondents who report identical EQ-5D-5L health profiles. Among these respondents, both educational attainment and, more prominently, income status were systematically associated with higher EQ VAS scores. These findings are consistent with residual health heterogeneity that is not reflected in the five EQ-5D-5L dimensions, and with the hypothesis of reporting heterogeneity by socioeconomic status. They do not imply that the EQ-5D-5L descriptive system fails to capture content it was designed to measure; rather, they describe the relationship between EQ-5D-5L responses and socioeconomic indicators not contained in the descriptive system.

## Supplementary Information

Below is the link to the electronic supplementary material.

Supplementary Material 1

## Data Availability

No datasets were generated or analysed during the current study.
