# Supplementary material for: Measuring inequality in quality of life: further evidence that the EQ-5D-5L may underestimate it
Source: Qual Life Res. 2026 Jun 15;35(8):199. doi: 10.1007/s11136-026-04294-w (PMC13269496; doi:10.1007/s11136-026-04294-w)
Supplement: Supplementary file 1 — Supplementary Material 1 [file 11136_2026_4294_MOESM1_ESM.docx]

**Supplementary Materials**

**Table S1. Description of frequent and moderate-level EQ-5D-5L profiles**

| **Profile** | **n (%)** | **Cum. Freq. (%)** | **EQ VAS mean (SD)** | **Excluded cases** |
| --- | --- | --- | --- | --- |
| *Most frequent profiles* | | | | |
| 11111 | 8832 (27.0) | 27.0 | 84.3 (14.3) | 263 |
| 11121 | 3353 (10.2) | 37.2 | 80.5 (13.2) | 30 |
| 11122 | 2471 (7.6) | 44.8 | 75.4 (14.2) | 36 |
| 21121 | 695 (2.1) | 46.9 | 78.0 (14.1) | 6 |
| 11112 | 2326 (7.1) | 54.0 | 78.0 (15.0) | 37 |
| 11221 | 309 (0.9) | 54.9 | 76.0 (14.1) | 2 |
| *Moderate-level profiles* | | | | |
| 11131 | 457 (1.4) | 56.3 | 71.5 (16.8) | 13 |
| 11132 | 360 (1.1) | 57.4 | 69.8 (14.3) | 5 |
| 21231 | 222 (0.7) | 58.1 | 64.2 (15.3) | 4 |
| 11123 | 888 (2.7) | 60.8 | 69.1 (15.5) | 19 |
| *Note.* For comparability with the previous study on a different dataset, we selected the same EQ‑5D‑5L profiles, although two of them (21121 and 11221) were less prevalent in the current dataset. SD: standard deviation. Respondents were excluded if they had body mass index (BMI) less than 15 kg/m², were younger than 25 years or older than 80 years, or if their EQ‑5D‑5L profile and EQ VAS score were judged internally inconsistent (EQ VAS <50 for profile 11111 and <30 for all other profiles). | | | | |

**Table S2. Country-stratified SEP gradient in EQ VAS within the full-health profile (11111), aged 25–79 years**

| **Variables** | **AUS** | **CAN** | **FRA** | **GER** | **NLD** | **NZL** | **UK** | **US** |
| --- | --- | --- | --- | --- | --- | --- | --- | --- |
| ***Panel A: Education gradient in EQ VAS*** | | | | | | | | |
| *Education (ref = Low)* |  |  |  |  |  |  |  |  |
| Medium | 0.085 | 1.901* | 4.016** | -0.348 | 0.453 | 0.677 | 2.785*** | 1.197 |
| (SE) | (1.065) | (1.116) | (1.610) | (0.865) | (0.704) | (1.048) | (1.045) | (1.214) |
| High | 1.876** | 0.565 | 3.287** | 0.536 | 0.440 | 3.083*** | 1.728** | 1.691** |
| (SE) | (0.813) | (0.948) | (1.435) | (0.627) | (0.892) | (0.875) | (0.760) | (0.854) |
| Male (ref = Female) | 1.168* | -0.653 | -1.879*** | -0.253 | -1.842*** | 0.742 | -1.260* | -1.809** |
| (SE) | (0.678) | (0.801) | (0.682) | (0.584) | (0.590) | (0.626) | (0.663) | (0.725) |
| Constant | 81.478*** | 85.636*** | 84.109*** | 88.022*** | 83.013*** | 83.223*** | 84.429*** | 83.990*** |
| (SE) | (1.386) | (1.691) | (1.921) | (1.209) | (1.199) | (1.394) | (1.373) | (1.453) |
| R-squared | 0.011 | 0.006 | 0.015 | 0.003 | 0.019 | 0.018 | 0.012 | 0.016 |
| ***Panel B: Income status gradient in EQ VAS*** | | | | | | | | |
| *Income (ref = Difficult)* |  |  |  |  |  |  |  |  |
| Coping | 3.065*** | 1.210 | 1.509 | 0.502 | 1.707* | 3.471*** | 1.218 | 1.374 |
| (SE) | (1.019) | (1.127) | (0.952) | (0.989) | (1.000) | (0.883) | (0.927) | (1.198) |
| Comfortable | 6.355*** | 2.437** | 3.534*** | 2.992*** | 4.508*** | 7.392*** | 3.681*** | 4.441*** |
| (SE) | (0.966) | (1.087) | (0.984) | (0.960) | (1.028) | (0.834) | (0.967) | (1.105) |
| Male (ref = Female) | 0.382 | -0.852 | -2.195** | -0.251 | -2.083*** | 0.393 | -1.505** | -1.687** |
| (SE) | (0.677) | (0.800) | (0.684) | (0.577) | (0.583) | (0.606) | (0.663) | (0.713) |
| Constant | 78.715*** | 85.180*** | 85.417*** | 86.309*** | 81.241*** | 81.179*** | 85.162*** | 82.434*** |
| (SE) | (1.381) | (1.629) | (1.456) | (1.409) | (1.270) | (1.194) | (1.260) | (1.556) |
| Observations | 1,232 | 724 | 1,068 | 1,181 | 1,234 | 1,156 | 1,129 | 845 |
| R-squared | 0.046 | 0.009 | 0.023 | 0.020 | 0.043 | 0.078 | 0.021 | 0.042 |
| *Note.* Entries are linear regression coefficients with robust standard errors in parentheses. Each column is a separate regression for the indicated country. Dependent variable: EQ VAS score. Panel A reports the education model; Panel B reports the corresponding income model. Country abbreviations: AUS (Australia), CAN (Canada), FRA (France), GER (Germany), NLD (Netherlands), NZL (New Zealand), UK (United Kingdom), US (United States). * *p* < 0.10; ** *p* < 0.05; *** *p* < 0.01. | | | | | | | | |

**Table S3. Country-stratified SEP gradient in EQ VAS within profile 11121, aged 25–79 years**

| **Variables** | **AUS** | **CAN** | **FRA** | **GER** | **NLD** | **NZL** | **UK** | **US** |
| --- | --- | --- | --- | --- | --- | --- | --- | --- |
| ***Panel A: Education gradient in EQ VAS*** | | | | | | | | |
| *Education (ref = Low)* |  |  |  |  |  |  |  |  |
| Medium | 1.033 | 3.243** | -1.103 | -2.788** | -0.481 | 0.952 | 1.335 | 4.445** |
| (SE) | (1.527) | (1.448) | (2.536) | (1.384) | (1.352) | (1.503) | (1.839) | (1.744) |
| High | 1.085 | 4.827*** | -0.938 | 1.773 | -1.400 | 2.909** | 2.191 | 5.173*** |
| (SE) | (1.240) | (1.433) | (2.343) | (1.147) | (2.015) | (1.438) | (1.582) | (1.427) |
| Male (ref = Female) | -2.411** | -2.299** | -0.465 | -2.144** | -1.203 | 0.513 | -1.341 | -0.892 |
| (SE) | (1.113) | (1.136) | (1.165) | (1.049) | (1.208) | (1.182) | (1.379) | (1.162) |
| Constant | 77.368*** | 74.635*** | 81.589*** | 83.821*** | 74.469*** | 70.028*** | 72.029*** | 73.338*** |
| (SE) | (2.509) | (2.783) | (3.366) | (2.286) | (2.677) | (2.346) | (3.254) | (2.544) |
| R-squared | 0.017 | 0.041 | 0.001 | 0.032 | 0.046 | 0.065 | 0.022 | 0.051 |
| ***Panel B: Income status gradient in EQ VAS*** | | | | | | | | |
| *Income (ref = Difficult)* |  |  |  |  |  |  |  |  |
| Coping | 4.249*** | 1.181 | 0.902 | 3.027* | 1.854 | 2.195 | 5.147*** | 0.418 |
| (SE) | (1.430) | (1.628) | (1.574) | (1.629) | (1.718) | (1.358) | (1.783) | (1.801) |
| Comfortable | 5.123*** | 2.941* | 3.432** | 6.894*** | 2.088 | 5.018*** | 8.047*** | 5.012*** |
| (SE) | (1.554) | (1.636) | (1.642) | (1.606) | (1.861) | (1.568) | (2.004) | (1.775) |
| Male (ref = Female) | -2.540** | -2.171* | -0.855 | -2.221** | -1.226 | 0.518 | -1.955 | -0.905 |
| (SE) | (1.097) | (1.148) | (1.169) | (1.030) | (1.205) | (1.180) | (1.351) | (1.156) |
| Constant | 76.600*** | 78.095*** | 79.727*** | 78.894*** | 72.236*** | 70.814*** | 73.284*** | 75.575*** |
| (SE) | (2.332) | (2.645) | (2.638) | (2.585) | (2.613) | (2.108) | (3.029) | (2.625) |
| Observations | 451 | 388 | 450 | 479 | 373 | 403 | 362 | 417 |
| R-squared | 0.042 | 0.023 | 0.013 | 0.059 | 0.048 | 0.078 | 0.059 | 0.057 |
| *Note.* Entries are linear regression coefficients with robust standard errors in parentheses. Each column is a separate regression for the indicated country. Dependent variable: EQ VAS score. Panel A reports the education model; Panel B reports the corresponding income model. Country abbreviations: AUS (Australia), CAN (Canada), FRA (France), GER (Germany), NLD (Netherlands), NZL (New Zealand), UK (United Kingdom), US (United States). * *p* < 0.10; ** *p* < 0.05; *** *p* < 0.01. | | | | | | | | |
